# Development of an African horse sickness VP6 DIVA diagnostic ELISA

**DOI:** 10.1186/s12985-025-02898-1

**Published:** 2025-08-12

**Authors:** Munyaradzi Tinarwo, Susan J. Dennis, Inga I. Hitzeroth, Ann E. Meyers, Edward P. Rybicki, Sandiswa Mbewana

**Affiliations:** 1 Biopharming Research Unit, Department of Molecular and Cell Biology, University of Cape Town, Rondebosch, Cape Town 7700 South Africa; 2 Institute of Infectious Disease and Molecular Medicine, University of Cape Town, Rondebosch, Cape Town 7701 South Africa

**Keywords:** African horse sickness virus, Molecular pharming, ELISA, DIVA, Diagnosis, VP6

## Abstract

**Background:**

African horse sickness (AHS) is a severe, noncontagious disease of equines caused by the African horse sickness virus (AHSV). The virus has nine serotypes and is transmitted by the *Culicoides* midge. AHS is endemic in South Africa and other sub-Saharan African countries. Currently, the disease is managed using a live attenuated vaccine manufactured by Onderstepoort Biological Products (OBP). Although this vaccine has been in use for decades, it has several drawbacks, including the possibility of reversion to virulence, and it does not allow for the differentiation of infected horses from vaccinated horses (DIVA). Previously, our group developed recombinant AHSV serotype 4 and 5 virus-like particle (VLP) vaccine candidates in plants that elicited an immune response in guinea pigs and horses. In this research, we aimed to develop a diagnostic enzyme-linked immunosorbent assay (ELISA) using an AHSV-VP6 antigen expressed in plants, allowing for the differentiation of horses infected with the virus from those vaccinated with VLP vaccine candidates. For this DIVA ELISA, we utilized a robust, cost-effective, and easily scalable manufacturing process that employs transient expression of VP6 in *Nicotiana benthamiana*.

**Results:**

AHSV-VP6 sequences from all nine serotypes were aligned to obtain a consensus sequence, which was then used to design the *VP6* gene. The *VP6* gene was successfully expressed in *Nicotiana benthamiana* plants via *Agrobacterium*-mediated infiltration. The VP6 protein was extracted from infiltrated leaves and purified. A purified yield of approximately 7.7 mg of recombinant VP6/kg fresh weight leaf material was obtained. The VP6 protein was also expressed in *E. coli*, yielding a purified product of 9.4 mg/L. Preliminary data revealed that AHSV-VP6 antigen expressed in both plants and *E. coli* could be used to differentiate between sera from infected horses and those vaccinated with the candidate AHSV4 and AHSV5 VLP vaccines. The plant-produced VP6 could detect more anti-VP6 antibodies than the *E. coli-*produced VP6.

**Conclusions:**

In this study, we expressed the AHSV-VP6 protein in plants, which enabled the differentiation of infected AHSV horse sera from those of horses vaccinated with the candidate VLP vaccines. To our knowledge, this is the first evidence of AHSV-VP6 expression in plants and the first demonstration of its diagnostic ability.

**Supplementary Information:**

The online version contains supplementary material available at 10.1186/s12985-025-02898-1.

## Background

African horse sickness (AHS) is a disease that affects equines and is considered to be the most lethal disease of horses. AHS is a noncontagious, notifiable disease allocated to List A status by the WHO OIE [1]. Mortality rates can reach 95%, with horses being the most affected species. Zebras are believed to be the main reservoir of the virus between outbreaks [2–4].

AHS has serious implications for a wide range of African communities. Equines play an important role in providing draft power for farmers in low-income communities, helping to alleviate poverty and food shortages [5]. On the other hand, horse racing and the export of horses to European countries and other parts of the world have extensive economic benefits, creating employment, tourism, and entertainment. Horses are also good companion animals [6, 7].

The etiological agent for AHS is the African horse sickness virus (AHSV), a member of the *Orbivirus* genus in the *Reoviridae* family that is closely related to Bluetongue virus (BTV), which affects ruminants such as sheep and cattle [4]. The AHSV genome consists of 10 linear double-stranded RNA segments encoding seven structural proteins (VP1-VP7) and four nonstructural proteins (NS1-NS4). AHSV is a nonenveloped icosahedral virus that is 70 nm in diameter and consists of three layers: the core (VP3 and VP7), the transcriptional complex (VP1, VP4, and VP6), and the outer capsid (VP2 and VP7) [2–4, 8].

There are nine different serotypes of AHSV, all of which have been isolated in South Africa [9]. The disease is endemic in sub-Saharan Africa, North Africa, the Middle East, the Arabian Peninsula, and the Mediterranean [10]. Direct transmission of AHSV is attributed to the *Culicoides* midge. The midge has always been present in Africa, but due to climate change, it has migrated slowly northward and has been reported in Europe. Recently, in 2020, an AHSV outbreak was recorded in Thailand; this was the first AHSV outbreak ever recorded in Southeast Asia and the first serotype 1 outbreak outside Africa [11, 12].

There is no cure for AHS apart from supportive therapy and good animal husbandry. AHS is mainly controlled through quarantining infected animals, movement restrictions during pandemics, vector control, and vaccination [2, 13]. In South Africa, a polyvalent live attenuated vaccine (LAV) manufactured by Onderstepoort Biological Products (OBP, Pretoria) is currently used to control the disease. Although the LAV has been effective for decades, its use continues to carry ongoing risks that warrant attention. These concerns include the possibility of reversion to virulence and gene segment reassortment between outbreak strains and vaccine strains, which could result in a new virus [14]. Additionally, the seed virus strains used originate from South Africa; therefore, their use elsewhere could lead to the introduction of new strains into different ecosystems. The vaccine is also not licensed for use in Europe, and one cannot distinguish between naturally infected and vaccinated animals (DIVA), which makes surveillance difficult and expensive [2, 3, 8, 14].

To overcome the challenges associated with current vaccine approaches, various recombinant strategies have been explored to develop efficacious, safe, inexpensive, and DIVA-compliant vaccines. VP2, the serotype determinant, elicits neutralizing antibodies and has been the major target antigen for developing these vaccines [15–18]. Strategies based on DNA [19], the poxvirus vector [20–22], reverse genetics [23–25], subunit vaccines [16, 26, 27], and VLP vaccines [28–32] have been explored in an effort to develop new AHS candidate vaccines.

The outer capsid VP7 protein is a group-specific antigen and is highly conserved among serotypes, which makes it the antigen of choice for the diagnosis of AHS [33]. Several diagnostic strategies have been explored, including viral isolation (virus neutralization and complement fixation) [34], antigen identification (direct immunofluorescence, in situ and blot hybridization [35], and reverse transcriptase‒polymerase chain reaction (RT‒PCR) [36, 37], and antibody identification (complement fixation, immunofluorescence, and ELISA) [1, 38]. Indirect ELISA for detecting the VP7 protein [39] and a VP7 blocking ELISA [26, 40, 41] are commercially available for AHSV. These two ELISA tests are recommended for the serological diagnosis of AHSV and assessment of equids before the international movement of animals [1].

Although the use of the LAV (OBP) in conjunction with VP7 ELISA has been effective in controlling and managing the disease, it does not allow for the differentiation of vaccinated animals from those infected with the virus. To address this challenge, candidate AHSV vaccines in the form of VLPs made in *Nicotiana benthamiana* have been investigated by our group. These VLPs, which are composed of four structural proteins, namely, VP2, VP3, VP5, and VP7, have been shown to be highly effective, eliciting an antibody response in guinea pigs and horses [29, 30, 32]. However, despite the possibility of reversion to virulence and gene segment reassortment between outbreak strains and vaccine strains, these vaccines still do not enable the distinction between infected and vaccinated animals because universal diagnostic assays rely on the detection of VP7. It would be desirable to develop a corresponding assay for VLP vaccine candidates that utilizes one of the AHSV proteins not present in the plant-produced VLPs. One of the three minor structural proteins, VP6, which is encoded by segment 9, is an example of this type of protein. It has a motif similar to helicases [42], which is known to be an early serological marker for AHS, making it an attractive candidate antigen for the rapid diagnosis of AHSV that allows for differentiation between infected and vaccinated horses [43].

The work herein describes the cloning and expression of AHSV VP6 in the plant *Nicotiana benthamiana*, its purification, and subsequent investigations into its potential use via indirect ELISA for detecting AHSV proteins.

## Methods

### Cloning of AHSV *VP6*

A total of 175 different AHSV *VP6* sequences from all 9 AHSV serotypes were downloaded from GenBank (accession numbers in Table S1) and aligned using CLC Main Workbench Software (Qiagen) to generate a consensus sequence that was synthesized (GenScript Biotechnologies, Piscataway, NJ, USA). The *VP6* gene was human codon-optimized after comparative analysis with the plant codon-optimized version, which revealed that the human version exhibited a more favorable GC content for expression. It also included a 6x histidine tag at the C-terminus, two restriction sites at the 5^’^ end (*Afl*III and *Age*I), and an *Xho*I at the 3^’^ end. When synthesized, the *VP6* gene was expected to be 1107 bp, and when expressed, it would yield a 369 amino acid protein (Figure S1). The *VP6* gene was directly cloned and inserted into three different plant expression vectors to test for optimal expression of the antigen: pRIC4.0 (autonomously replicating vector) [44], pTRAc (targeting protein to expression) [45], and pEAQ-HT (hyperexpression vector) [46] via *Afl*III or *Age*I and *Xho*I restriction enzymes. These vectors were selected to compare and identify the most efficient for expressing the VP6 protein in plants, as they differ in their expression cassettes and regulatory elements. *VP6* was also cloned and inserted into the bacterial expression vector pProEx-HTc (Invitrogen™, California, USA) via the *Afl*III and *Xho*I restriction sites. The recombinant constructs pRIC4.0-*VP6*, pTRAc-*VP6*, pEAQ-HT-*VP6*, and pProEx-HTc-*VP6* were transformed into *E. coli* cells, and their presence was confirmed by colony PCR using vector-specific primers (listed in Table S2) and restriction enzyme mapping. Plasmid DNA from the AHSV-*VP6* constructs was isolated from the transformed *E. coli* cells via a QIAprep Spin Miniprep Kit (Qiagen, Germany). Cloning was further confirmed by digesting the purified plasmids with either *Afl*III or *Xho*I (pRIC4.0-*VP6*, pTRAc-*VP6*, and pProEx-HT-*VP6*) or *Age*I and *Xho*I (pEAQ-HT-*VP6*). The digested DNA samples were fractionated on 1% (w/v) Tris-borate-EDTA (TBE) (89 mM Tris base, 89 mM boric acid and 2 mM EDTA) agarose gels containing 2.5 mg/mL ethidium bromide and visualized under short-wavelength (260 nm) illumination to verify the presence and expected sizes of the insert and vector backbone fragments.

### Agrobacterium tumefaciens transformation

*A. tumefaciens* GV3101::pMp90RK and AGL-1 (ATCC. BAA101™) strains were obtained from the Biopharming Research Unit culture collection (University of Cape Town). Both *A. tumefaciens* strains were prepared for electroporation as previously described [47]. *A. tumefaciens* GV3101::pMp90RK was electroporated with 100 ng of pRIC4.0-*VP6*, pTRAc-*VP6*, or AGL-1 with pEAQ-HT-*VP6* using a GenePulser (Bio-Rad, California, USA). The recombinant strains were selected on LB media plates supplemented with 25 µg/ml carbenicillin and 50 µg/ml kanamycin (pEAQ-HT constructs) and 50 µg/ml carbenicillin, 30 µg/ml kanamycin, and 50 µg/ml rifampicin (pRIC4.0 and pTRAc constructs). Successful transformation of the cells was confirmed via colony PCR using vector-specific primers (Table S2).

### *Agrobacterium*-mediated infiltration of VP6 in *N. benthamiana* plants

VP6 protein expression in the plants was assessed via a small-scale three-day time trial over 2–5 days post infiltration (dpi) at three different optical densities (OD_600_) of 0.25, 0.5, and 1.0. The recombinant *A. tumefaciens* transformants were inoculated into Luria Bertani-based broth (LBB)-enriched media (0.25% tryptone, 1.25% yeast extract, 0.5% NaCl, and 10 mM 4-morpholineethanesulfiric acid, pH 5.6) and incubated at 27 °C overnight with shaking at 180 *rpm*. The cultures were subcultured as previously described [48], and on the final day, the cultures were diluted to the desired OD_600_ in resuspension buffer (5 mM 4-morpholineethanesulfiric acid, 20 mM MgCl_2_, 0.2 mM acetosyringone, pH 5.6). Three five- to six-week-old *N. benthamiana* plants per OD_600_ per construct were vacuum infiltrated for small-scale expression optimization by submerging the plants into the bacterial suspension and applying a vacuum of between 90 and 95 kPa. The plants were then grown in a plant room under optimum conditions (22–25 °C, 16-h/8-h light/dark cycles).

### Transient expression analysis of VP6 in plants

The infiltrated leaves were harvested from 3 to 5 dpi. The VP6 protein was extracted from 2 g of infiltrated leaves by homogenization in 1× PBS extraction buffer (137 mM NaCl, 10 mM Na_2_HPO_4_, 2.7 mM KCl, 2 mM KH_2_PO_4_, pH 7.4] containing Complete™, EDTA-free protease inhibitor cocktail [Roche, Basel, Switzerland]) at a ratio of 1:2 (w/v) with a T25 digital ULTRA-TURRAX^®^ homogenizer (Sigma‒Aldrich^®^, St. Louis, MO, USA). The plant extract was clarified by centrifugation at 13000 *rpm* for 20 min using a JA-14 (Beckman Coulter Inc., Brea, CA) rotor and an Avanti^®^ J25RI centrifuge (Beckman).

The concentration of total soluble protein (TSP) in the crude extract was determined by Bradford assay using bovine serum albumin (BSA) (Sigma-Aldrich, MO, USA) as a standard. The extracts were boiled for 10 min in sample application buffer (SAB) (2% SDS, 100 mM Tris-HCl, pH 7.5, 2 mM EDTA, 52% glycerol, 4.3% β-mercaptoethanol, 0.25% bromophenol blue). Equal volumes of TSP (5 µg) were loaded onto 10% sodium dodecyl sulfate (SDS)-polyacrylamide gels and separated by electrophoresis at 120 V for 2 h. The gels were electroblotted onto a 0.45 μm nitrocellulose membrane (Amersham Protran, USA) at 15 V for 1 h and 30 min using a Trans-blot^®^ SD semi-dry blotter (Bio-Rad, California, USA). The recombinant VP6 protein was detected by a 1:2000 dilution of anti-histidine antibody (Bio-Rad, California, USA). The blot was probed with a secondary alkaline phosphatase-conjugated anti-mouse antibody (Abcam, Cambridge, United Kingdom) at a 1:10 000 dilution. The VP6 protein was detected by nitro blue tetrazolium chloro/5-bromo-4 chloro-3-indolyl phosphate (NBT/BCIP) substrate (KPL, Gaithersburg, MD, USA).

### Expression of the recombinant VP6 protein in *E. coli*

The *E. coli* transformants containing pProEx-HTc-VP6 were inoculated into LB media supplemented with 100 µg/mL ampicillin and incubated at 37 °C with agitation at 230 *rpm*. The cultures were grown until the OD_600_ reached between 0.6 and 1.0. The cultures were induced with 0.6 mM IPTG (isopropylthio-β-D-galactoside) and further incubated for 1, 2, or 3 h to determine the optimal expression time.

Large-scale VP6 *E. coli* expression was achieved by growing the culture in 500 ml of LB media and incubating as described previously until the OD_600_ reached between 0.6 and 1.0. The culture was then induced with 0.6 mM IPTG, and the cells were collected after 3 h by centrifugation at 8073 *rpm* for 10 min.

### Purification of the recombinant VP6 protein

For large-scale expression of the recombinant VP6 protein, leaves from 40 infiltrated *N. benthamiana* plants were homogenized, clarified 3 times by centrifugation at 13 000 *rpm* for 20 min at 4 °C and filtered through 2 layers of Miracloth™ (EMD Millipore Corp., Billerica, MA, USA).

The clarified plant-crude leaf extract was then semi-purified by ammonium sulfate precipitation. Optimization of ammonium sulfate precipitation was performed with a series of increasing (NH_4_)_2_SO_4_ concentrations (0–20%, 20–30%, 30–40%, 40–50%, 50–60%, and 60–80%). The EnCor Biotechnology Inc. (USA) ammonium sulfate calculator tool available at https://www.encorbio.com/protocols/AM-SO4.htm was used to determine the amount of (NH_4_)_2_SO_4_ to add. (NH_4_)_2_SO_4_ was slowly added to the clarified protein, and the sample was stirred for 30 min at 4 °C. The VP6 protein was then pelleted by centrifugation at 13,000 *rpm* for 20 min at 4 °C. The precipitated pellets were resuspended in half the original volume of buffer (50 mM Na_2_HPO_4_, 0.3 M NaCl, pH 7.0) and dialyzed overnight in 5 L of the same buffer at 4 °C using 10 kDa dialysis tubing (Thermo Fischer Scientific, USA). The *E. coli*-produced VP6 protein was also semi-purified from the cell pellet following the BugBuster (Merck, Germany) protocol according to the manufacturer’s instructions. The pellet was dialyzed in the same way as the plant-produced VP6.

Prior to histidine affinity purification, the semi-purified VP6 (plant or *E. coli*-produced) was filtered through a 0.45 μm filter (GVS, Italy). The protein was purified with an automated fast protein liquid chromatography (FPLC) system (AKTA Purifier Plus, GE Healthcare Life Technologies). A flow rate of 2.5 min/ml was used for sample application on a column equilibrated with binding buffer (50 mM Na_2_HPO_4_, 0.3 M NaCl, and 10 mM imidazole, pH 7.0). The column was washed with 10 column volumes of wash buffer (50 mM Na_2_HPO_4_, 0.3 M NaCl, and 30 mM imidazole, pH 7.0). VP6 was eluted with 5 column volumes of elution buffer (50 mM Na_2_HPO_4_, 0.3 M NaCl, 0.5 M imidazole, pH 7.0).

The eluted fractions were separated by 10% SDS‒PAGE gel electrophoresis as described above and subsequently stained with Coomassie blue (0.1% Brilliant Blue R-250, 50% methanol, and 10% glacial acetic acid). Fractions in which the VP6 protein was detected with higher purity were pooled together and concentrated using a 30 kDa Amicon^®^ Ultra15 centrifuge tube (Merck Millipore, Ireland). The concentrated VP6 protein was then quantified using gel densitometry. The optical intensities of the Coomassie blue-stained gel were determined using a Gene Genius Bioimaging System GeneTools (version 3.07.03) (Synegene). Bovine serum albumin (BSA) (Roche Diagnostics, Germany) was used as a standard to measure the concentration of the VP6 protein.

### Preparation of mock antigen

Mock antigen was prepared by infiltration of *Agrobacterium tumefaciens* carrying an empty plant expression vector (pRIC4.0). The processing and purification of mock antigen from infiltrated leaves were carried out as described above. *E. coli* cells transformed with the pProEx-HTc vector lacking an insert were used to prepare mock *E. coli* antigen in the same manner as for the *E. coli-*produced VP6 described above.

### Indirect AHSV-VP6 ELISA

Indirect ELISA was employed to determine whether the recombinant AHSV-VP6 protein could detect anti-VP6 antibodies in serum from animals that had been vaccinated with the LAV and in serum from those vaccinated with the plant-produced AHSV-4 and − 5 VLP candidate vaccines [30, 32]. Ninety-six-well MaxiSorp^®^ microtiter plates were coated in triplicate with 100 µL/well containing 100 ng of either plant or *E. coli*-produced VP6 protein diluted in coating buffer (10 mM Tris, pH 8.5). The plate was incubated overnight at 4 °C with agitation. The plate was blocked with 200 µL of Tris-buffered saline (TBS) blocking buffer (3% fat-free milk in 1X TBS [50 mM Tris, 150 mM NaCl, pH 7.5]) for 1 h at 37 °C. The serum from the horses was diluted 1:500 in TBS blocking buffer, 100 µL was added to each well, and the plate was incubated at 37 °C for 1 h, after which it was washed four times with 200 µL of TST buffer (1X TBS, 0.05% Tween^®^20, pH 7.5). One hundred microliters of anti-horse IgG alkaline phosphatase conjugate (Sigma, Aldrich, USA) diluted 1:5000 in blocking buffer was added to each well, and the plate was incubated at 37 °C for 1 h. Following incubation, the ELISA plate was washed four times with 200 µL of 1XTBS buffer (50 mM Tris, 150 mM NaCl, pH 9.0). Each sample was detected by adding 200 µL of SIGMAFAST™ p-nitrophenyl phosphate (pNPP, Sigma) per well, and the plate was incubated for 30 min in darkness at room temperature. Optical density (OD_405_) values were measured using a BIOTEK^®^ Powerwave XS microtiter plate reader. The OD_405_ readings were converted to a percentage of positive control serum (PP) using the following equation:

$$\begin{array}{*{20}{l}}{\:{\bf{Percentage}}\:{\bf{PP}}}\\{ = \frac{\begin{array}{l}{\rm{Mean}}\:{\rm{OD}}\:{\rm{of}}\:{\rm{test}}\:{\rm{sample}}\:\\- \:{\rm{Mean}}\:{\rm{OD}}\:{\rm{of}}\:{\rm{negative}}\:{\rm{control}}\end{array}}{\begin{array}{l}{\rm{Mean}}\:{\rm{OD}}\:{\rm{of}}\:{\rm{positive}}\:{\rm{control}}\\\: - \:{\rm{Mean}}\:{\rm{OD}}\:{\rm{of}}\:{\rm{negative}}\:{\rm{control}}\end{array}}}\\{ \times 100\% }\end{array}$$

adopted from [49].

## Results

### Generation of AHSV-VP6-expressing constructs

**Fig. 1:**
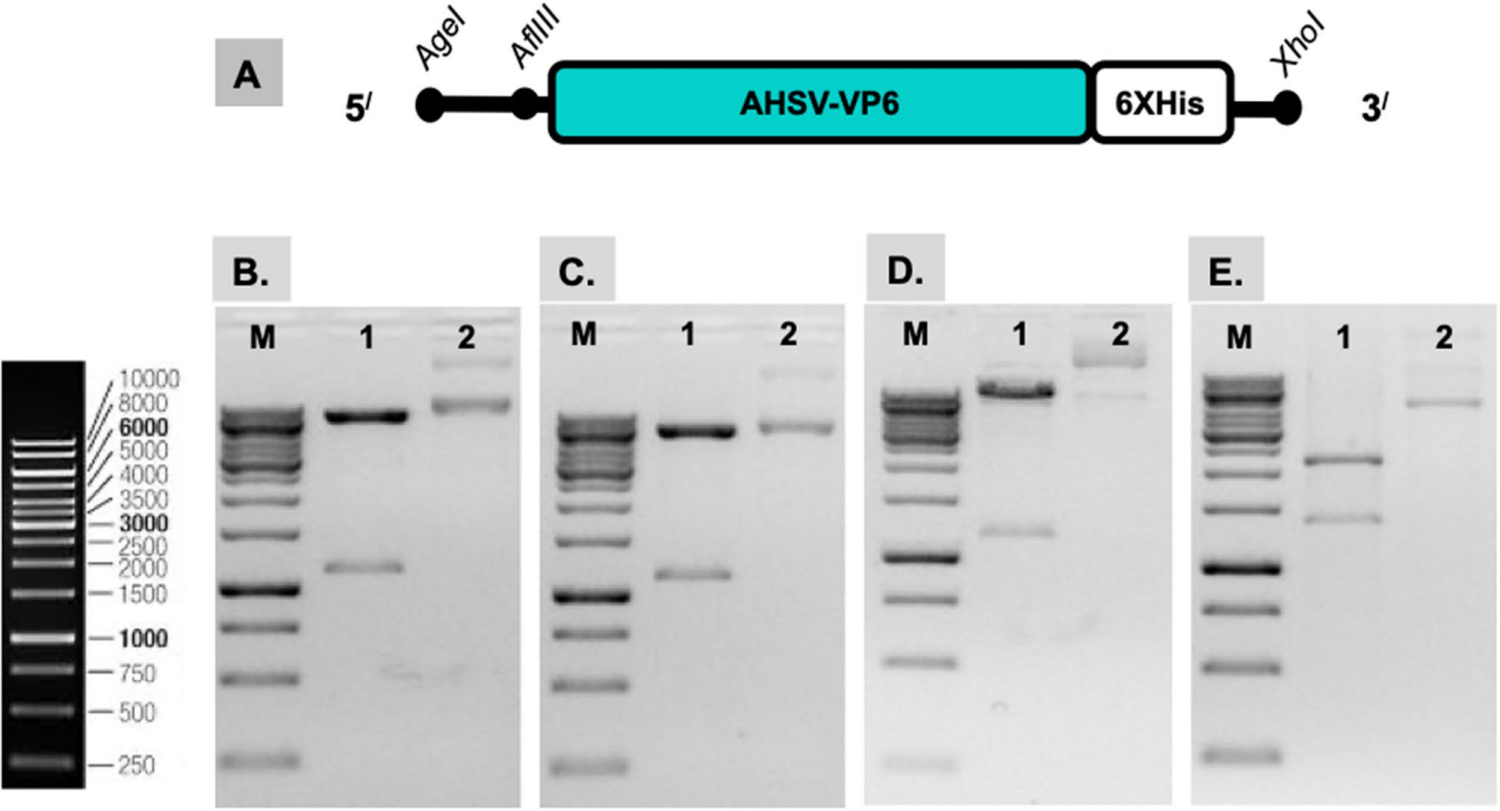
Confirmation of AHSV-VP6 constructs through restriction mapping digestion. **A**. Schematic representation of the synthesized AHSV-*VP6*. **B-D.** Restriction mapping of the AHSV-VP6 constructs. **M** GeneRuler 1 kb DNA Ladder (Thermo Fisher Scientific, Lithuania) served as a DNA marker; **lane 1** shows the plasmid that was digested with restriction enzymes, while **lane 2** displays the undigested plasmid resolved on a 1% (w/v) TBE agarose gel. **(B)** pRIC4.0-VP6 (8479 bp) was digested with *Afl*III and *Xho*I to yield fragments of 7349 bp and 1130 bp. **(C)** pTRAC-VP6 (7277 bp) was digested with *Afl*III and *Xho*I to yield fragments of 6147 bp and 1130 bp. **(D)** pEAQ-HT-VP6 (11,085 bp) was digested with *Age*I and *Xho*I to yield fragments of 9949 bp and 1136 bp. **(E)** pProExHTc-VP6 (5832 bp) was digested with *Afl*III and *Xho*I to yield fragments of 2241 bp, 2218 bp, and 1373 bp

The *VP6* gene, illustrated in Fig. 1a, was synthesized and received in the pUC57 vector. *VP6* was cloned and inserted into three plant expression vectors to create pRIC4.0-*VP6*, pTRAc-*VP6*, and pEAQ-HT-*VP6*, aiming to select the construct that would result in the highest accumulation of the VP6 protein in *N. benthamiana* for further investigation. Additionally, the *VP6* gene was cloned and inserted into the pProExHTc vector to generate pProExHTc-VP6 for the expression of VP6 in *E. coli* as a control. Successful clones were confirmed by colony PCR (results not shown) and subsequent restriction enzyme mapping of the plasmids purified from selected transformants (Fig. 1b-d). Four constructs were developed for *VP6* expression in both plants and *E. coli*.

### Expression of VP6 in *N. benthamiana* and *E. coli*

**Fig. 2:**
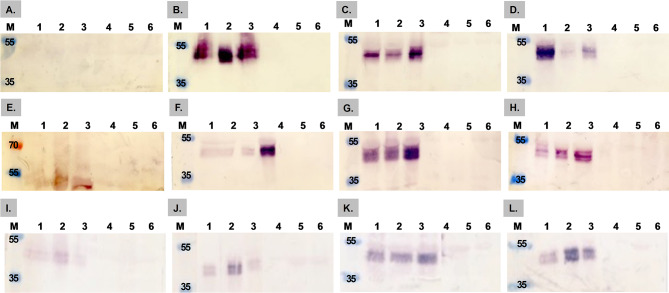
Expression time trial of the AHSV-VP6 constructs in *N. benthamiana* plants. Western blot analysis was conducted using an anti-histidine primary antibody. **A–D**: pRIC4.0‒VP6 from 2–5 dpi; **E–H**: pTRAc-VP6 from 2–5 dpi; **I–L**: pEAQ-HT-VP6 from 2–5 dpi. **Lanes 1–3** depict crude leaf extracts from leaves infiltrated at OD600 values of 0.25, 0.5, and 1.0, respectively; **Lanes 4–6** exhibit mock-infiltrated crude leaf extracts at OD600 values of 0.25, 0.5, and 1.0, respectively. **Lane M** contains a PageRulerTM Plus Prestained protein ladder (Thermo Scientific, Lithuania)

Leaves from *N. benthamiana* plants infiltrated with the pRIC4.0-*VP6*, pTRAc-*VP6*, and pEAQ-HT-*VP6* constructs were harvested, and crude leaf extracts underwent western blot analysis. An expression time trial from 2 to 5 dpi for the three constructs was performed. No expression of the 43 kDa VP6 protein was detected at 2 dpi with the pRIC4.0-*VP6* and pTRAc-*VP6* constructs; however, low expression was observed with the pEAQ-HT-*VP6* construct (Fig. 2A, E, and **I)**. The VP6 expression from pRIC4.0-VP6 was high at 3 dpi, decreasing by 5 dpi for OD_600_ values of 0.5 and 1.0, while the expression level at OD_600_ 0.25 increased from 3 dpi and peaked at 5 dpi (Fig. 2B, C, and **D**). The expression levels of pTRAc-VP6 rose from 3 to 4 dpi but declined by 5 dpi, with the highest expression detected at 4 dpi. Compared to other OD_600_ values, the plants infiltrated at an OD_600_ of 1.0 exhibited greater expression (Fig. 2F, G, and **H**). The expression levels of pEAQ-HT-VP6 gradually increased from 2 to 5 dpi, with the highest levels observed at 5 dpi in plants infiltrated at an OD_600_ of 0.5 (Fig. 2I–L). To reduce the time and the OD_600_ used to produce VP6, the crude leaf extracts from the three constructs at 3 dpi with OD_600_ values of 0.25 and 0.5 were compared (Figure S2). As expected, no expression of the VP6 protein was detected in the crude leaf extracts from plants infiltrated with empty vectors (pRIC4.0, pTRAc, and pEAQ-HT), which served as negative controls (Fig. 2, lanes 4–6).

The highest protein accumulation of VP6, based on the banding intensity observed in the western blots, was identified in leaves infiltrated with cells harboring pRIC4.0-VP6 (Figure S2). Furthermore, the *Agrobacterium* culture OD_600_ of 0.5, harvested at 3 dpi, resulted in better expression than the other OD_600_ values and dpi values. Thus, pRIC4.0-VP6 was selected for further investigation using an infiltration concentration of 0.5 and harvesting leaves at 3 dpi. Interestingly, VP6 appeared as a doublet (approximately 43 and 42 kDa) in all expressed constructs (Figure S2). Compared to the lower band, the upper VP6 band exhibited greater intensity. Analysis of our VP6 sequence was conducted using SnapGene (GSL Biotech LLC, Chicago, IL, USA) bioinformatics software, identifying two potential translation products: a 39.2 kDa product in the reading frame + 1 (375 amino acids, 1128 bp, with a 6x histidine tag at the C-terminus) and a 38 kDa product in the reading frame − 2 (372 amino acids, 1119 bp, without a 6x histidine tag, though it contains a high number of histidine amino acids). Some post-translational modifications may have occurred, resulting in two distinct products. Further investigations would be required to identify the cause of this doublet.

**Fig. 3:**
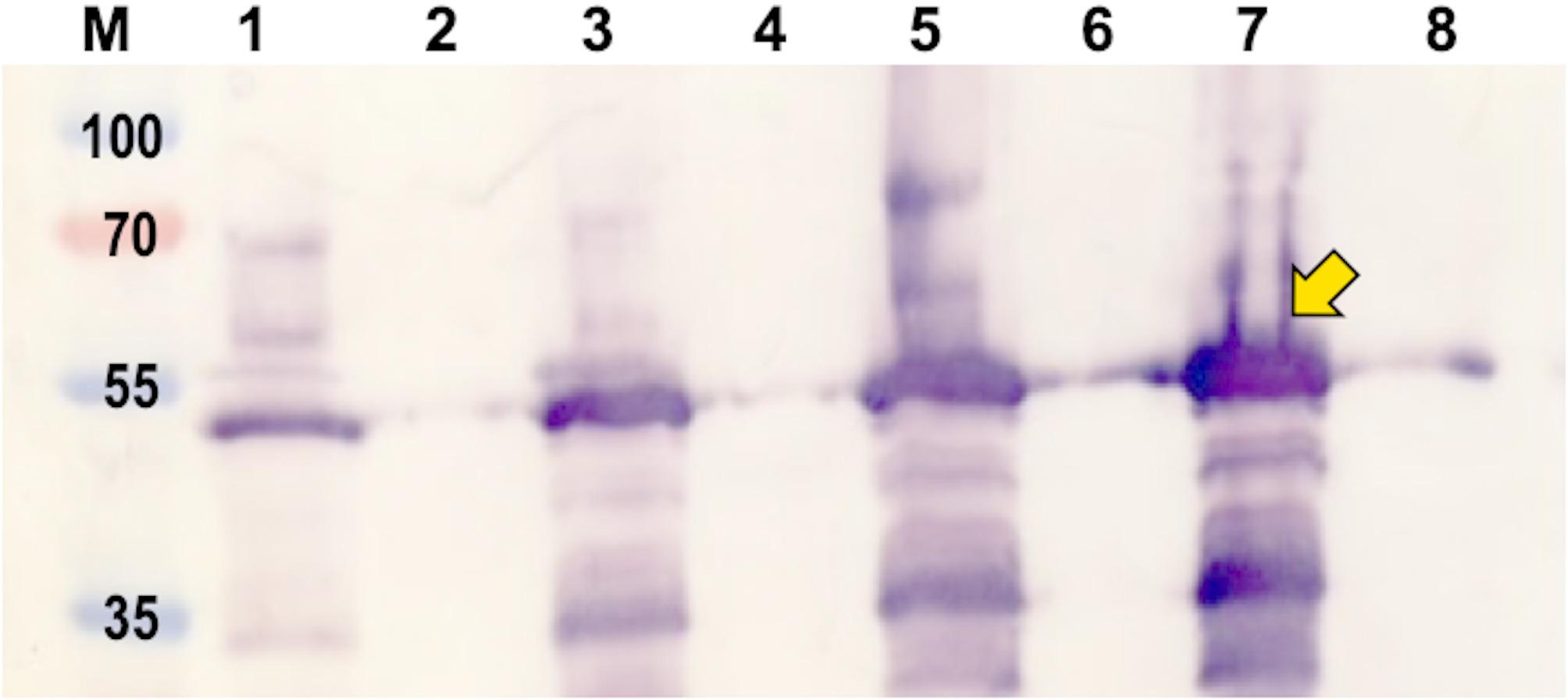
Expression analysis of the VP6 *antigen* produced in *E. coli*. Optimization of the VP6 antigen expression in *E. coli involved a* time trial to determine the optimal duration for expression following IPTG induction. The pellet of the cells (**lane 1**) and their supernatant (**lane 2**) are shown before IPTG induction. **Lanes 3** and **4** depict the pellet and supernatant, respectively, 1 h after induction. **Lanes 5** and **6** show the pellet and supernatant, respectively, 2 h after induction. **Lanes 7** and **8** illustrate the pellet and supernatant, respectively, 3 h following induction. **Lane M** contains a PageRuler™ Plus Prestained protein ladder (Thermo Scientific, Lithuania). The yellow arrow highlights the highest expression of VP6 based on band intensity

The expression of VP6 in *E. coli* using the pProEx-HTc-VP6 construct was examined during a small-scale time trial with IPTG induction. VP6 expression was detected by Western blot, revealing a 43 kDa protein band that was observed before IPTG induction and increased after 1, 2, and 3 h of post-induction (Fig. 3). For large-scale VP6 production in *E. coli*, an optimal duration of 3 h was selected for future experiments, as it yielded better expression (Fig. 3, lane 7) than the other tested time points based on band intensity. The VP6 protein was predominantly detected in the pelleted cells, with trace amounts observed in the centrifuged culture supernatant. Expression from an empty pProEx-HTc vector served as a negative control (data not shown). Interestingly, in contrast to the plant-produced VP6, no doublet was observed from the *E. coli* expression of VP6.

### Purification of the recombinant VP6 protein

**Fig. 4:**
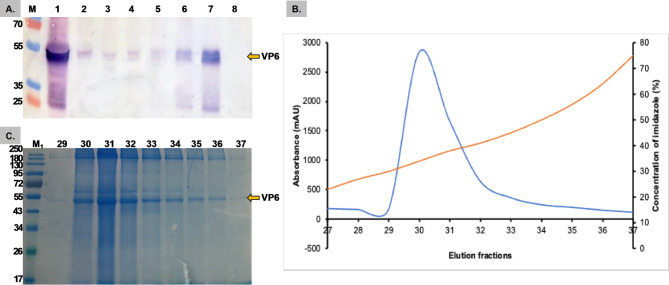
Nickel resin purification of the (NH_4_)_2_SO_4−_ precipitated plant-produced VP6. **(A)** Optimization of ammonium sulfate saturation for the plant-produced VP6. Western blot analysis: **Lane M** contained the PageRuler™ Plus Prestained protein ladder (Thermo Scientific, Lithuania), **lane 1** contained the crude leaf extract; **lanes 2–6** contained the precipitates from 20–60% ammonium sulfate in 10% increments; **lane 7** contained the precipitates from 60–80% ammonium sulfate; and **lane 8** contained the empty pRIC4.0 vector control. **(B)** Chromatographic trace showing the elution of the plant-produced VP6 (blue) from the affinity column with increasing imidazole concentration (red). **(C)** Coomassie blue-stained SDS‒PAGE of the elution fractions [29–37] and the **M**_**1**_ color prestained protein standard ladder (New England Biolabs)

The production of the VP6 protein in plants was scaled up, and the crude leaf extract was semi-purified. Western blot analysis of the ammonium sulfate-precipitated fractions revealed that the VP6 protein was detected in all fractions, but most precipitated in the 60–80% (NH_4_)_2_SO_4_ fraction (Fig. 4a). The semi-purified VP6 protein was further purified using histidine affinity chromatography, with the protein eluted in fractions 29–37 from the column at increasing concentrations of imidazole (Fig. 4b). The eluted fractions were qualitatively assessed by SDS‒PAGE and Coomassie blue staining for purity (Fig. 4c). They were deemed satisfactory for use as an antigen in an ELISA due to the presence of few contaminating proteins. The highest accumulation of the VP6 protein was detected in fractions 30–31; however, fractions 34–37 appeared to contain fewer contaminants, so these were pooled and quantified. The average yield from three trials was calculated to be approximately 7.77 mg/kg of fresh-weight leaf material.

**Fig. 5:**
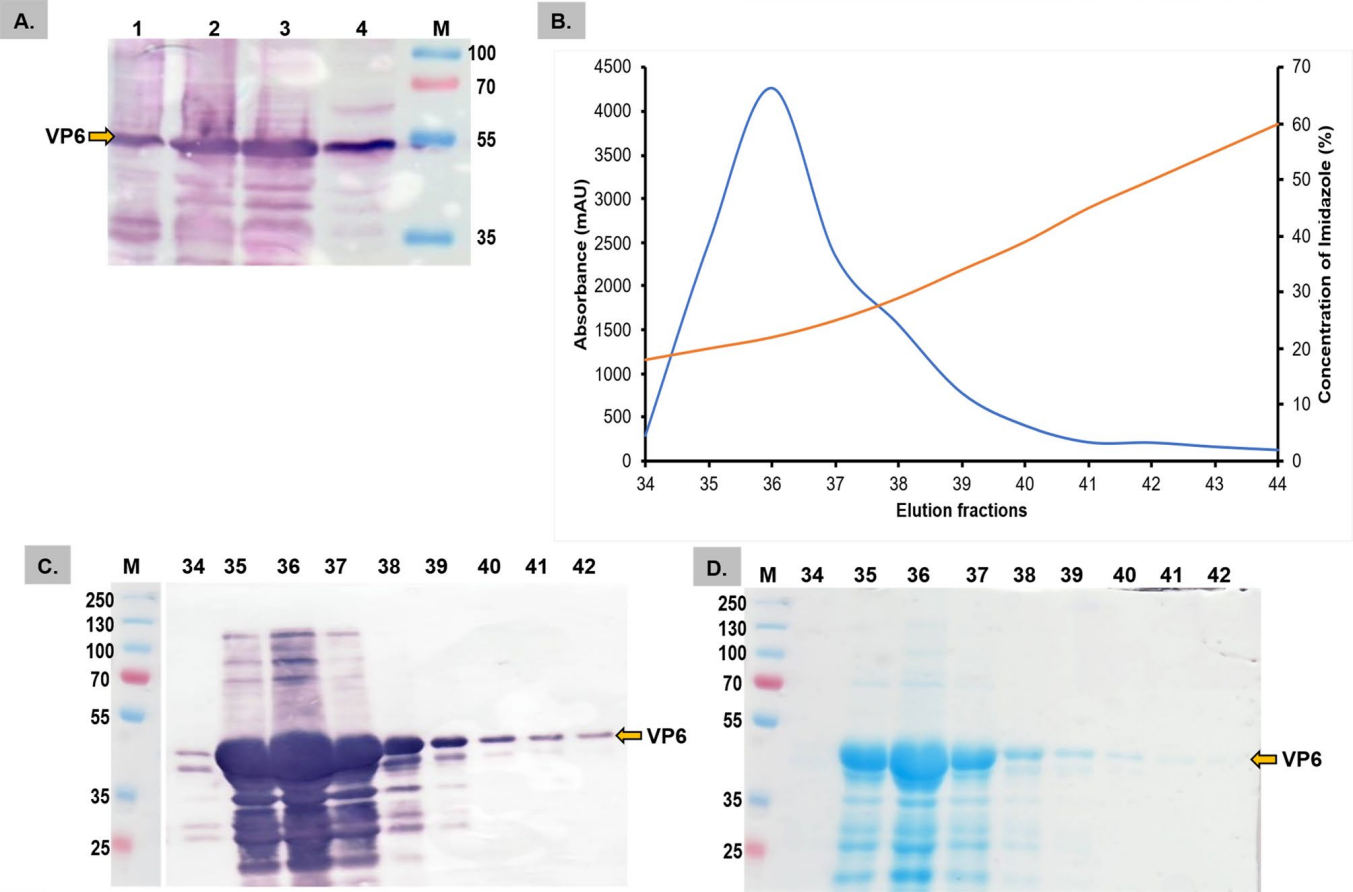
Nickel resin purification of the semi-purified *E. coli-* produced VP6 protein. **(A)** Western blot analysis of large-scale semi-purification of the VP6 protein using the BugBuster purification protocol. **Lane 1** represents the pelleted cells from the culture, **lane 2** represents the cell lysate (after treatment with benzonase), **lane 3** represents the BugBuster VP6 soluble fraction, and **lane 4** represents the BugBuster VP6 insoluble fraction. **(B)** Chromatographic trace showing the elution of *E. coli*-produced VP6 (blue) from the affinity column with increasing imidazole concentration (red). **(C)** Western blot analysis of the eluted fractions [34–42], probed with the anti-histidine antibody. **(D)** Corresponding Coomassie blue-stained SDS‒PAGE, with **lane M** containing the PageRuler™ Plus Prestained protein ladder (Thermo Scientific, Lithuania)

The *E. coli*-expressed VP6 was produced on a large scale. The VP6 protein was found to be partially soluble, as it was detected mostly in the soluble fraction (supernatant) rather than in the insoluble fraction (pellet) (Fig. 5a). The supernatant was subsequently dialyzed and purified using nickel‒histidine affinity column chromatography, and the VP6 protein was eluted from fractions 34–42 (Fig. 5b) with increasing concentrations of imidazole. The eluted fractions were evaluated by western blotting, SDS‒PAGE, and Coomassie blue staining (Fig. 5c and **d**). The highest accumulation of the VP6 protein occurred in fraction 36, with good expression also observed in fractions 35 and 37. Based on the purity shown on the Coomassie-stained gel, fractions 39–42 appeared to contain fewer contaminants and were therefore pooled together and quantified. The yield from three trials was calculated to be approximately 9.4 mg/L of bacterial culture.

### Indirect ELISA using VP6

The ability of the purified plant- and *E. coli*-produced VP6 proteins to distinguish between the presence or absence of VP6-specific antibodies in sera from horses vaccinated with either the plant-produced AHSV 4 and 5 VLPs or those vaccinated with the LAV vaccine was assessed. An indirect ELISA using both plant- and *E. coli*-produced VP6 as the coating antigen was performed to evaluate its DIVA functionality. A plant-produced empty pRIC4.0 vector, purified in the same manner as the VP6 antigen, served as a mock antigen (data not shown). The serum concentration used in the ELISA was optimized to 1:500 by titration. The cutoff value to differentiate infected sera from vaccinated animals was calculated as the percentage of positive control serum (PP) values exceeding 15.0. The PP values of LAV sera were 49.11 and 21.01 when the plant- or *E. coli*-produced VP6 was used, respectively, indicating that the plant-produced antigen detected more anti-VP6 antibodies than the *E. coli*-produced VP6 antigen. The PP values of sera from naïve and VLP-vaccinated horses ranged from 3.31 to 14.82 and from 4.52 to 10.82, respectively, when using the plant or *E. coli*-produced VP6 antigen.

**Fig. 6:**
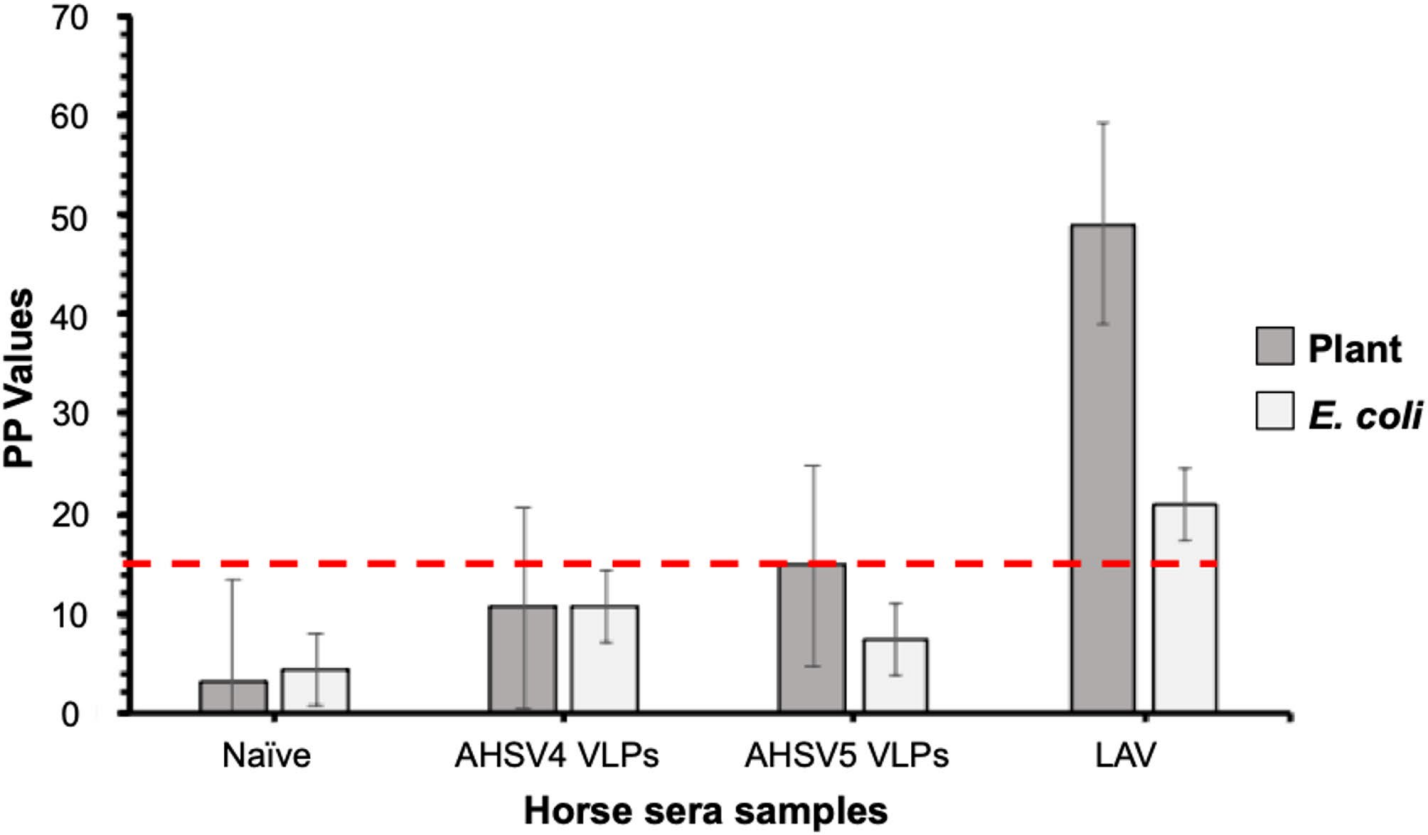
Analysis of the DIVA diagnostic functionality of both plant- and *E. coli*-produced VP6. This graph illustrates the capability of plant- and *E. coli*-produced VP6 to detect anti-VP6 antibodies in sera from naïve horses, sera from horses vaccinated with plant-produced AHSV4 and AHSV5 VLPs, and sera from horses vaccinated with the LAV vaccine. Horse serum was tested at a 1:500 dilution. The black bars represent the mean PP values for the plant-produced VP6 antigen, while the gray bars illustrate the *E. coli*-produced VP6 antigen. The error bars depict the standard deviation of the PP value. The cutoff value (red line) to differentiate between the presence and absence of anti-VP6 antibodies in the serum was established at 15

## Discussion

AHSV is a devastating disease of horses, with mortality rates reaching as high as 95%. The disease is endemic in sub-Saharan Africa, but due to climate change, there have also been reports of the virus in Europe [10], and most recently, the first outbreak of AHSV was recorded in Thailand in 2020 [12]. This outbreak was the first occurrence in Southeast Asia and the first detection of serotype 1 outside Africa [11]. AHSV is currently controlled by vaccination with a live attenuated vaccine produced by Onderstepoort Biological Products. However, this vaccine has drawbacks, such as not allowing differentiation between infected and vaccinated animals. Recently, in our group (Biopharming Research Unit at UCT), we developed candidate AHSV serotype 4 and 5 VLP vaccines comprising the VP2, VP3, VP5, and VP7 antigens in plants. The candidate VLP vaccines were found to be immunogenic; however, they still do not facilitate distinguishing between vaccinated and infected horses because the universally accepted diagnostic assay utilizes VP7 as the detecting antigen, which is itself a component of the plant-produced VLPs. It was deemed prudent to investigate the possibility of using VP6 as an alternative diagnostic antigen, which could enhance the use of plant-produced VLP vaccines [29, 30, 32].

In this study, we investigated the potential of using the AHSV-VP6 protein expressed in either *N. benthamiana* or *E. coli* as an antigen to differentiate between horses infected with AHSV and those vaccinated with plant-produced AHSV VLP vaccine candidates. The VP6 antigen (43 kDa) was expressed in both systems and detected by western blot analysis (Figs. 2 and 3). The plant-produced VP6 appeared as a doublet on the western blot, similar to the results obtained when VP6 from BTV serotype 1 was expressed in vitro and in insect cells [50]. Conversely, the *E. coli*-produced VP6 appeared as a single band, consistent with the results in the literature [51]. Bioinformatics analysis of our VP6 sequence revealed two possible translation products, although the smaller one lacks the 6x histidine tag. This likely corresponds to the lower 42 kDa VP6 band, which shows low intensity on the western blot. Despite lacking the 6x histidine tag, this translation product contains 27 histidine residues compared to the 6 histidine residues (plus the histidine tag) in the + 1 ORF, most of which are located near the N-terminus. It is possible that these proteins could fold and bind to anti-histidine antibodies as well as to the resin during purification, explaining their detection via a western blot probed with anti-His antibodies.

The VP6 antigen was successfully purified using anti-histidine affinity chromatography (Figs. 4 and 5). The production of the VP6 protein in *E. coli* yielded approximately 9.4 mg/L bacterial culture. A yield of soluble plant-produced VP6 antigen of 7.77 mg/kg of fresh-weight leaf material was obtained, which is higher than yields of other plant-expressed diagnostic antigens [52–54]. On the basis of the yield of the VP6 protein extracted and the concentration of the VP6 protein needed to test one sample, approximately 100 g of infiltrated leaves can theoretically be used to test up to 3,200 serum samples. These findings prove that this AHSV-VP6 DIVA diagnostic ELISA is potentially less expensive, which makes it affordable and suitable for low- and medium-income countries.

The capability of the VP6 antigens (produced in both plants and *E. coli*) to serve as diagnostic antigens differentiating between infected and vaccinated horses was demonstrated using indirect ELISA. The recombinant VP6 antigens successfully distinguished serum from horses vaccinated with the live attenuated vaccine (which mimics infected sera) from serum of horses vaccinated with the plant-produced VLP vaccines (Fig. 6). The PP values for the sera from horses vaccinated with the LAV were 21.01 and 49.11 when testing *E. coli* and plant-produced VP6, respectively. These values exceeded the PP values for sera from horses vaccinated with the AHSV 4 and 5 VLP vaccine candidates, ranging from 10.57 to 14.82. The PP values from naïve horses were low, at 3.31 (plant) and 4.52 (*E. coli*). Based on the absorbances obtained, the VP6 expressed in plants bound to more anti-VP6 antibodies than that expressed in *E. coli*, demonstrating superior DIVA capability. This may be attributed to the plant expression system’s ability to allow for correct protein folding, resulting in enhanced functionality of the antigen [55]. To reduce the possibility of non-specific binding of the antibodies and lower the cut-off PP values, the purification process can be refined using size exclusion chromatography.

In this study, serum from horses vaccinated with the live attenuated AHSV vaccine was used instead of serum from infected horses due to restrictions on working with live virus or infected samples in the Western Cape Province, South Africa, where our laboratory is located [14]. Ideally, sera from horses infected with AHSV should have been used in this study, but since the LAV elicits antibodies against all AHSV antigens and this research serves as a proof-of-concept, the absence of infected horse sera should not be viewed as a limitation. Currently, there is a need for an ELISA test that can detect antibodies not present in the vaccine. The plant-produced VLP-based vaccines consist entirely of structural proteins. They do not contain VP6. Incorporating VP6 in the assay, alongside these vaccines, will ensure DIVA compliance.

The serum sample size was limited to the number of horses vaccinated during the immunogenicity testing of the candidate VLP vaccines. Although this study demonstrated that VP6 can be used to differentiate infected from vaccinated horse sera, further validation of DIVA ELISA functionality with a larger sample size is needed and harmonized across laboratories. VP6 could also be evaluated as a diagnostic antigen in other field-applicable assays, such as a lateral flow assay. Additionally, there is a need to validate the use of the VP6 ELISA and compare it to the current AHSV ELISAs available on the market, including the INGEZIM AHSV COMPAC PLUS 2.0 (Ingenesa, Spain) and the ID Screen African Horse Sickness Indirect ELISA (Innovative Diagnostics, France), to determine its sensitivity and specificity in a larger pool of samples. To our knowledge, this is the first time that the AHSV-VP6 protein has been expressed in plants. Its ability to differentiate between infected animals and those vaccinated with the candidate AHSV-4 and AHSV-5 VLP vaccines shows promise.

## Supplementary Information

Below is the link to the electronic supplementary material.

Supplementary Material 1

Supplementary Material 2

## Data Availability

No datasets were generated or analysed during the current study.

## Abbreviations

AHS: African horse sickness
AHSV: African horse sickness
OBP: Onderstepoort Biological Products
DIVA: Differentiation of infected horses from vaccinated horses
VLP: Virus-like particle
ELISA: Enzyme-linked immunosorbent assay
BTV: Bluetongue virus
LAV: Live attenuated vaccine
RT-PCR: Reverse transcriptase‒polymerase chain reaction
TSP: Total soluble protein
VLP: Virus-like particle
ELISA: Enzyme-linked immunosorbent assay
BTV: Bluetongue virus
LAV: Live attenuated vaccine
