# Supplementary material for: Development of an African horse sickness VP6 DIVA diagnostic ELISA
Source: Virol J. 2025 Aug 12;22:276. doi: 10.1186/s12985-025-02898-1 (PMC12344834; doi:10.1186/s12985-025-02898-1)
Supplement: Supplementary file 1 — Supplementary Material 1 [file 12985_2025_2898_MOESM1_ESM.docx]

**Development of an African horse sickness VP6 diagnostic ELISA**

**SUPPLEMENTARY FILE: UNCROPPED GELS AND BLOTS IMAGE(S)**

**NOTE:** All the parts of the gels that were cropped out are indicated with the red box

**Figure 1. Confirmation of AHSV-VP6 constructs through restriction mapping digestion.** **A**. Schematic representation of the synthesized AHSV-*VP6*. **B – D.** Restriction mapping of the AHSV-VP6 constructs. **M** GeneRuler 1 kb DNA Ladder (Thermo Fisher Scientific, Lithuania) served as the DNA ladder; **lane 1** shows the plasmid digested with restriction enzymes, and **lane 2** displays the undigested plasmid resolved on a 1% (w/v) TBE agarose gel. **B.** pRIC4.0-VP6 (8479 bp) was digested with *Afl*III and *Xho*I to yield fragments of 7349 and 1130 bp. **C.** pTRAC-VP6 (7277 bp) was digested with *Afl*III and *Xho*I to yield fragments of 6147 and 1130 bp. **D.** pEAQ-HT-VP6 (11,085 bp) was digested with *Age*I and *Xho*I to yield fragments of 9949 and 1136 bp. **E.** pProExHTc-VP6 (5832 bp) was digested with *Afl*III and *Xho*I to yield fragments of 2241, 2218, and 1373 bp.

**Figure 2: Expression time trial of the AHSV-VP6 constructs in *N. benthamiana* plants.** Western blot analysis was conducted using an anti-histidine primary antibody. **A–D);** pRIC4.0‒VP6 from 2-5 dpi; **E–H);** pTRAc-VP6 from 2–5 dpi **(I–L);** pEAQ-HT-VP6 from 2–5 dpi. Lanes 1–3 correspond to crude leaf extracts from leaves infiltrated at OD_600_ values of 0.25, 0.5, and 1.0, respectively, while **Lanes 4–6** correspond to mock-infiltrated crude leaf extracts at OD_600_ values of 0.25, 0.5, and 1.0, respectively. **Lane M** contains a PageRuler^TM^ Plus Prestained protein ladder (Thermo Scientific, Lithuania).

**Figure 3. Expression analysis of the *E. coli*-produced VP6 antigen.** Optimization of VP6 antigen expression in *E. coli*. A time trial was conducted to determine the optimal time for VP6 antigen expression after induction with IPTG. The pellet of cells (**lane 1**) and their supernatant (**lane 2**) are shown before IPTG induction. **Lanes 3** and **4** represent the pellet and supernatant, respectively, 1 hour after induction. **Lanes 5** and **6 depic**t the pellet and supernatant, respectively, 2 hours after induction. **Lanes 7** and **8 illustrate** the pellet and supernatant, respectively, 3 hours after induction. **Lane M** contains a PageRuler^™^ Plus Prestained protein ladder (Thermo Scientific, Lithuania). The yellow arrow indicates the highest VP6 expression based on band intensity.

**Figure 4. Nickel resin purification of the (NH_4_)_2_SO_4-_precipitated plant-produced VP6. A.** Optimization of ammonium sulfate saturation for the plant-produced VP6. Western blot analysis: **lane M** contained the PageRuler™ Plus Prestained protein ladder (Thermo Scientific, Lithuania), **lane 1** included the crude leaf extract, **lanes 2–6 includ**ed the precipitates from 20% to 60% ammonium sulfate in 10% increments, **lane 7** included the precipitates from 60% to 80% ammonium sulfate, and **lane 8** contained the empty pRIC4.0 vector control. **B.** Chromatographic trace depicting the elution of the plant-produced VP6 (blue) from the affinity column as imidazole concentration increased (red). **C.** Coomassie blue-stained SDS‒PAGE of the elution fractions (**29-37**) and **M_1_** color prestained protein standard ladder (New England Biolabs).

**Figure 5. Nickel resin purification of the semi-purified *E. coli* produced VP6 protein. A.** Western blot analysis of the large-scale semi-purification of the VP6 protein using the BugBuster purification protocol. **Lane 1** represents the pelleted cells from the culture, **lane 2** represents the cell lysate (after treatment with benzonase), **lane 3** represents the BugBuster VP6 soluble fraction, and **lane 4** represents the BugBuster VP6 insoluble fraction. **B.** Chromatographic trace showing the elution of *E. coli*-produced VP6 (blue) from the affinity column as the imidazole concentration increases (red). **C.** Western blot analysis of the eluted fractions (34-42) probed with anti-histidine antibody. **D.** Corresponding Coomassie blue-stained SDS-PAGE, where **lane M** contains the PageRuler^™^ Plus Prestained protein ladder (Thermo Scientific, Lithuania).

**Figure 6. Analysis of the DIVA diagnostic functionality of the plants and *E. coli-*produced VP6.** This graph illustrates the capability of both plant- and *E. coli*-produced VP6 to detect anti-VP6 antibodies in sera from naïve horses, as well as in sera from horses vaccinated with plant-produced AHSV4 and AHSV5 VLPs, and sera from horses vaccinated with the LAV vaccine. Horse serum was used at a 1:500 dilution. The black bars indicate the mean PP values for the plant-derived VP6 antigen, while the gray bars represent the *E. coli*-derived VP6 antigen. The error bars denote the standard deviation of the PP values. The cutoff value (red line) for distinguishing between the presence or absence of anti-VP6 antibodies in the serum was established at 15.

**Figure S2. Expression analysis of the VP6 antigen using the plant expression system.** Transient expression time trial of the VP6 protein in *N. benthamiana* by *Agrobacterium-*mediated transfer. The three different plant expression vectors (pEAQ-HT, pRIC4.0 and pTRAc) were infiltrated at OD_600_ values of 0.25 and 0.5 at 3 dpi. **Lane M** contains a PageRuler^TM^ Plus Prestained protein ladder (Thermo Scientific, Lithuania). The gold arrow indicates the highest expression of VP6 at an OD_600_ of 0.5, the green arrow indicates the highest VP6 doublet band, whereas the red arrow indicates the lowest band.
