# Supplementary material for: Development of an African horse sickness VP6 DIVA diagnostic ELISA
Source: Virol J. 2025 Aug 12;22:276. doi: 10.1186/s12985-025-02898-1 (PMC12344834; doi:10.1186/s12985-025-02898-1)
Supplement: Supplementary file 2 — Supplementary Material 2 [file 12985_2025_2898_MOESM2_ESM.docx]

# Supplementary data

**Table S1:** GenBank accession numbers for the AHSV-VP6 sequences for all nine serotypes that were aligned to generate the consensus sequence that was eventually used to create the AHSV-VP6 gene

| AHSV serotype | Number of sequences | GenBank accession numbers | | | | | |
| --- | --- | --- | --- | --- | --- | --- | --- |
| 1 | 54 | AQV08636 | AQV08626 | AQV08616 | AQV08606 | ALL62730 | ALL62720 |
|  |  | ALL54938 | ALL43346 | ALL43326 | ALL43296 | AKP19969 | ALL62710 |
|  |  | ALL54958 | ALL54948 | ALL54928 | ALL54908 | ALL54898 | ALL54888 |
|  |  | ALL54878 | ALL54868 | ALL54848 | ALL54838 | ALL54688 | ALL54678 |
|  |  | ALL54668 | ALJ92410 | AKP19967 | AKP19963 | AQV08596 | ALL54818 |
|  |  | ALL54808 | ALL54788 | ALL54768 | ALL54748 | ALL54738 | ALL54728 |
|  |  | ALL54718 | ALL54708 | ALL54698 | ALL43356 | ALL43336 | ALL43306 |
|  |  | ALL43316 | ALL54918 | ALL54858 | ALL54778 | AKP19962 | ALL54758 |
|  |  | ALL54968 | AKP20110 | AKP19966 | AKP19965 | AKP19964 | AKP19968 |
| 2 | 3 | ALG63768 | AKP20039 | AKP20038 |  |  |  |
| 3 | 3 | AKP20019 | AKP20100 | AKP20018 |  |  |  |
| 4 | 8 | AKP39982 | ALL54798 | AKP19752 | AKP19753 | AKP20090 | AKP19754 |
|  |  | ALJ92420 | ALL54828 |  |  |  |  |
| 5 | 3 | AKP19833 | AKP19832 | AKP19834 |  |  |  |
| 6 | 8 | AKP19864 | ALG63778 | YP_052964 | ALG63798 | ALG63788 | AKP19862 |
|  |  | ALG63768 | AKP19863 |  |  |  |  |
| 7 | 4 | AKP19913 | AKP19912 | ALG63788 | AKP19914 |  |  |
| 8 | 6 | AKP19793 | AKP19791 | AKP19792 | AKP19790 | ALG63798 | AKP19794 |
| 9 | 86 | ALL62730 | ALL62720 | ALL54938 | ALL43346 | ALL43326 | ALL43296 |
|  |  | AKP19969 | ALL62710 | ALL54958 | ALL54948 | ALL54928 | ALL54908 |
|  |  | ALL54898 | ALL54888 | ALL54878 | ALL54868 | ALL54848 | ALL54838 |
|  |  | ALL54688 | ALL54678 | ALL54668 | ALJ92410 | AKP19967 | AKP19963 |
|  |  | ALL43356 | ALL43336 | ALL43306 | ALL43316 | ALL54918 | ALL54858 |
|  |  | ALL54818 | ALL54808 | ALL54788 | ALL54768 | ALL54748 | ALL54738 |
|  |  | ALL54728 | ALL54718 | ALL54708 | ALL54698 | ALL54778 | AKP20018 |
|  |  | AKP19962 | ALL54968 | ALL54758 | AKP20110 | AKP19914 | ALG63798 |
|  |  | AKP19834 | ALG63788 | AKP19833 | AKP39982 | ALL54798 | AKP19912 |
|  |  | AKP19966 | AKP19832 | AKP19790 | AKP20038 | AKP19913 | AKP19793 |
|  |  | AKP19791 | AKP19862 | AKP19792 | AKP19752 | AKP19864 | ALG63778 |
|  |  | YP_052964 | ALG63768 | AKP20039 | AKP19753 | AKP19794 | AKP20019 |
|  |  | AKP20100 | AAB17103 | ADI87401 | AKP19889 | AKP19888 | ALJ92420 |
|  |  | ALL54828 | AKP19968 | AKP20090 | AKP19754 | AFK24724 | AKP19863 |
|  |  | AKP19965 | AKP19964 |  |  |  |  |

**Table S2:** The sequences of the vector-specific primers and the corresponding PCR conditions

| Vector | Primer | Primer sequence (5^/^ - 3^/^) | Annealing temperature | | | |
| --- | --- | --- | --- | --- | --- | --- |
|  |  |  | **Condition** | **Temperature** | **Time** | **Number of cycles** |
| pTRAc and pRIC4.0 | forward | CATTTCATTTGGAGAGGACACG | Initial denaturation | 95$℃$ | 5 min | 1 |
|  |  |  | Denaturation | 95$℃$ | 30 sec | 30 |
|  |  |  | Annealing | 56$℃$ | 30 sec |  |
|  | reverse | GAACTACTCACACATTATTCTGG | Elongation | 72$℃$ | 30 sec |  |
|  |  |  | Final elongation | 72$℃$ | 5 min | 1 |
| pEAQ-HT | forward | TTCTTCTTCTTGCTGCTT | Initial denaturation | 95$℃$ | 5 min | 1 |
|  | reverse | CACAGAAAACCGCTCACC | Denaturation | 95$℃$ | 30 sec | 30 |
| pProEx-HTc | forward | CGTACTACCATCACCATCAC | Annealing | 55$℃$ | 30 sec |  |
|  | reverse | TGATTTAATCTGTATCAGGCTG | Elongation | 72$℃$ | 30 se |  |
|  |  |  | Final elongation | 72$℃$ | 5 min | 1 |

**Figure S1: AHSV-VP6 sequence**

SALLLAPGDLIEKRELEQRSITPLLREKDSKKSKLKEDGEKKNKSESEENKIHDDRERGESQKSSGSADCQRGAGSAGADCATSTGGGDGGAGARTGIGGVGGVDSRSGGHGGQGAASDGKGVGKSKTGADRVANDDATRNVGSSEVSSGGITSGGLQGRGGLVAKSSECGGEPLDRTGGCSGNSKTEGEEAKVGGGDRRIGGLATQEIADFVKKKIGVEVQVFSKGMSNLFTVDKSLLERGGLGREDILHQSDIVKEIRVSDKKVKIIPLSTVKRMIAEFGGTEEDEIKAVQTQSSSIRYISMEDVSRAKAMFTAPTGDEGWKEVAKAATQRPNIMAYVHEGDGLKELLHLIDHI

**Figure S2. Expression analysis of the VP6 antigen using the plant expression system.** Transient expression time trial of the VP6 protein in *N. benthamiana* was conducted through *Agrobacterium-*mediated transfer. The three different plant expression vectors (pEAQ-HT, pRIC4.0, and pTRAc) were infiltrated at OD_600_ values of 0.25 and 0.5 at 3 dpi. **Lane M** contains a PageRuler^™^ Plus Prestained protein ladder (Thermo Scientific, Lithuania). The gold arrow indicates the highest expression of VP6 at an OD_600_ of 0.5, the green arrow indicates the highest VP6 doublet band, while the red arrow indicates the lowest band.
